# Gene, microbiome, and anti-aging doping in sport: molecular mechanisms, detection strategies, and implications for sports rehabilitation medicine

**DOI:** 10.3389/fspor.2026.1984895

**Published:** 2026-10-09

**Authors:** Diego Cotella

**Affiliations:** Department of Health Sciences, Università del Piemonte Orientale, Novara, Italy

**Keywords:** anti-aging gene therapy, faecal microbiota transplantation, gene doping, gut microbiome, klotho, myostatin, sports rehabilitation, telomerase

## Abstract

Advances in gene transfer have created legitimate opportunities for tissue repair, but the same tools raise the prospect of illicit use to enhance athletic performance (“gene doping”). Evidence that elite athletes harbour a distinctive gut microbiota has raised a parallel concern that microbiome manipulation, including faecal microbiota transplantation (FMT) from high-performing donors, could be used as a form of biological enhancement (“microbiome doping”). A third, related frontier is anti-aging gene therapy (telomerase and Klotho gene delivery and partial cellular reprogramming) which targets the same muscle- and stem-cell-regenerative pathways relevant to injury recovery and has already reached unregulated human self-experimentation. This narrative review summarises the principal gene-doping targets relevant to performance and recovery (erythropoietin, myostatin/activin inhibitors, insulin-like growth factor 1, PPAR-δ/AMPK reprogramming), their delivery vectors, and current detection strategies. It then reviews athlete-associated gut microbiota signatures, notably the lactate-metabolising bacterium *Veillonella atypica*, and the feasibility and detection challenges of microbiota transplantation, before examining the rationale, targets, and governance challenges of anti-aging gene therapy. Attention is given to the overlap between all three frontiers and legitimate regenerative, microbiota-directed, or aging-biology therapies under investigation for musculoskeletal, metabolic, and gastrointestinal conditions, an overlap that places rehabilitation clinicians in a sensitive position, particularly with older or master athletes; across all three frontiers, the same biological pathways and delivery technologies are simultaneously being developed for legitimate therapeutic use, creating a recurring dual-use dilemma for clinicians and regulators alike. I review safety risks, the regulatory framework administered by the World Anti-Doping Agency (WADA) and its gaps regarding microbiome and longevity interventions, and the practical responsibilities of rehabilitation professionals. I conclude that interdisciplinary collaboration between molecular biologists, microbiome and aging researchers, anti-doping scientists, and clinicians is essential to keep detection methods and clinical governance aligned with the pace of innovation in these three areas, and we outline specific priorities, including clarification of the regulatory status of microbiota transfer and anti-aging gene constructs, strain-level metagenomic monitoring, and biological-age biomarkers, for achieving this alignment.

## 1 Introduction

Gene therapy (the transfer of genetic material into cells to treat or prevent disease) has moved from experimental promise to approved clinical practice over the past two decades, with licensed products now available for conditions ranging from spinal muscular atrophy to inherited retinal disease and certain haematological cancers. The same vector systems, delivery technologies, and gene targets that make these therapies effective also make them attractive, at least in principle, to individuals seeking to enhance muscle mass, endurance, or recovery capacity beyond what training and legal supplementation allow. The World Anti-Doping Agency (WADA) has classified this category of misuse as “gene and cell doping” since 2003, prohibiting the use of nucleic acids, nucleic-acid analogues, gene-editing agents, and normal or genetically modified cells with the potential to enhance sport performance (1, 2).

For clinicians and researchers working in sports rehabilitation, gene doping is not merely a distant anti-doping curiosity. Several of the biological pathways under investigation as doping targets (myostatin inhibition, growth-factor delivery, and pro-regenerative cytokine expression) are the very pathways being explored as legitimate treatments to accelerate healing after tendon, ligament, muscle, and cartilage injury (3). This dual-use character means that rehabilitation professionals may be among the first to encounter patients or athletes interested in, or already exposed to, unapproved genetic interventions, and that the field's own therapeutic innovations must be developed and communicated in ways that do not blur the line between medical treatment and performance enhancement. The same dual-use concern extends to two closely related and increasingly discussed frontiers: the deliberate manipulation of the athlete gut microbiome, including transplantation of microbiota from high-performing donors, and anti-aging gene therapy targeting the same muscle- and stem-cell-regenerative pathways, which has already reached unregulated human self-experimentation. This review summarises the current state of knowledge on gene-doping mechanisms and detection, extends this analysis to microbiome-based and anti-aging gene interventions, and discusses their combined relevance to sports rehabilitation practice.

Although gene doping, microbiome-based enhancement, and anti-aging gene therapy have each been reviewed separately, their convergence has received little systematic attention. The novelty of this review lies in treating these three frontiers as a single, interconnected phenomenon, unified by overlapping molecular pathways (notably myostatin/follistatin and pro-regenerative signalling), shared delivery technologies, and, most importantly for the intended readership, a common dual-use dilemma for sports rehabilitation clinicians who may encounter any of the three in clinical practice.

This review has three aims: first, to summarise the molecular targets, delivery systems, and detection strategies relevant to gene doping; second, to examine the emerging evidence for microbiome-based performance enhancement and its regulatory ambiguities; and third, to evaluate anti-aging gene therapy as a converging frontier with direct implications for sports rehabilitation practice. Across all three areas, I highlight the dual-use dilemma (the fact that the same biological pathways and technologies are being developed for legitimate therapeutic purposes) and consider its consequences for clinical governance, athlete education, and anti-doping policy.

This narrative review synthesised evidence from peer-reviewed literature on gene doping, microbiome-based performance enhancement, and anti-aging gene therapy. Searches were conducted in PubMed/MEDLINE, Google Scholar, and the websites of the World Anti-Doping Agency and the U.S. Food and Drug Administration for material published between 1998 and 2026, using combinations of the terms “gene doping”, “gene transfer, “erythropoietin gene”, “myostatin”, “follistatin”, “IGF-1”, “PPAR-δ”, “AMPK”, “CRISPR”, “gut microbiota”, “Veillonella atypica”, “faecal microbiota transplantation”, “microbiome doping”, “anti-aging gene therapy”, “telomerase”, “Klotho”, “partial reprogramming”, and “WADA Prohibited List”. Reference lists of retrieved articles were screened for additional relevant sources, and preprints and non-peer-reviewed sources (patents, regulatory safety communications, and reputable science-news reports) were included only where no peer-reviewed alternative existed and are identified as such in the text. As a narrative rather than systematic review, no formal inclusion/exclusion algorithm, dual screening, or risk-of-bias assessment was applied; source selection reflects the author's judgement of relevance and is a limitation discussed in Section 12.

## 2 Definition, history, and regulatory framework

WADA's Prohibited List defines gene and cell doping (Section M3) as the use of polymers of nucleic acids or nucleic acid analogues, gene editing agents that may alter genome sequences and/or the transcriptional or epigenetic regulation of gene expression, and normal or genetically modified cells, when these have the potential to enhance performance (2). The category has been part of the Prohibited List since it was first raised as a concern by the WADA Gene Doping Panel, following early demonstrations that muscle-directed gene transfer could produce dramatic phenotypic effects in animal models (1).

A landmark case that catalysed anti-doping attention to this field was the 2004 description of a child with a naturally occurring loss-of-function mutation in the myostatin (MSTN) gene, who presented with pronounced muscle hypertrophy and reduced fat mass (4). Although unrelated to doping, this case demonstrated in humans what animal experiments had already shown: that manipulating a single gene pathway could produce muscle phenotypes of obvious interest to strength and power athletes, and it accelerated both academic and anti-doping interest in myostatin-pathway manipulation. To the authors’ knowledge, and as of the time of writing, no confirmed case of gene doping in competitive sport has been publicly documented to date, but WADA-funded research programmes have continued to develop and validate detection assays in anticipation of its emergence, on the assumption that detection capability should precede, rather than follow, misuse (5).

## 3 Molecular targets and mechanisms

Most discussion of gene doping centres on a small number of well-characterised pathways with plausible performance-enhancing effects and pre-existing gene-therapy vectors developed for legitimate indications (6, 7).

### 3.1 Erythropoietin (EPO) gene transfer

Erythropoietin stimulates red-blood-cell production and increases oxygen-carrying capacity, making it attractive for endurance sports; recombinant EPO protein doping has been detectable for years through blood and urine testing. Gene-based EPO delivery (inserting an EPO-encoding sequence via a viral vector so that the body produces its own supraphysiological EPO continuously) was investigated experimentally as a hypothetical doping method as early as the mid-2000s (the so-called “Repoxygen” construct, originally developed for anaemia, became a recurring reference point in anti-doping literature after reports that a coach had attempted to source it for an athlete) (1, 2). Because gene-based EPO expression would bypass the typical pharmacokinetic pattern of injected recombinant EPO, it poses a distinct detection challenge and has therefore been a long-standing priority for assay development.

### 3.2 Myostatin/activin pathway inhibition

Myostatin (growth differentiation factor 8) is a member of the TGF-β superfamily that normally restrains skeletal-muscle growth; loss-of-function mutations or pharmacological/genetic inhibition of myostatin signalling produce muscle hypertrophy and hyperplasia in animal models and in the rare human cases described above (4, 8). Because myostatin inhibition also appears to influence connective-tissue remodelling, oxidative muscle phenotype, and muscle-stem-cell (satellite-cell) behaviour, it has attracted interest both as a doping target for strength athletes and as a candidate therapeutic strategy for muscle-wasting conditions and for accelerating recovery after musculoskeletal injury, including experimental work on myostatin blockade to limit quadriceps atrophy after anterior cruciate ligament (ACL) injury (9).

### 3.3 Insulin-like growth factor 1 (IGF-1) and other growth factors

Locally delivered IGF-1 and related growth factors (e.g., vascular endothelial growth factor, hepatocyte growth factor) promote muscle regeneration and angiogenesis and have been explored in animal models both for their capacity to accelerate recovery after injury and, in the anti-doping literature, as candidate performance-enhancing gene transfer agents given their trophic effects on muscle mass and repair capacity (3).

### 3.4 Metabolic reprogramming: PPAR-δ and AMPK pathways

Preclinical work on peroxisome proliferator-activated receptor delta (PPAR-δ) and AMP-activated protein kinase (AMPK) signalling demonstrated that pharmacological or genetic activation of these pathways can shift skeletal muscle toward a more oxidative, fatigue-resistant fibre phenotype and improve endurance capacity in animal models, giving rise to the informal label “exercise in a pill.” These pathways are monitored by anti-doping authorities as plausible future gene-doping targets for endurance sport, although evidence of translation to genetic manipulation in humans for this purpose remains lacking (7). The attractiveness of the PPAR-δ/AMPK axis, and of ACE and ACTN3 more generally, as gene-doping targets reflects their well-established role as naturally occurring genetic determinants of endurance and power phenotypes in athletes, independent of any doping context (10).

### 3.5 Genome-editing tools

The maturation of CRISPR-Cas and other programmable nuclease systems has, in principle, widened the toolkit available for genetic enhancement beyond simple gene addition to direct editing or epigenetic modulation of endogenous genes such as MSTN. WADA's current Prohibited List language was updated specifically to capture gene-editing agents and epigenetic modulation, reflecting this concern, even though evidence of gene-editing-based doping in athletes has not been documented (2, 7). Three related but mechanistically distinct classes of editing tool are relevant here. Base editors use a catalytically impaired Cas nuclease fused to a deaminase to convert one DNA base to another without generating a double-strand break, offering a theoretically lower off-target and genotoxicity profile than conventional CRISPR-Cas9 editing and thus a harder analytical target for detection assays that rely on break-repair signatures. Prime editors extend this “search-and-replace” approach using a reverse-transcriptase-Cas9 fusion and a prime-editing guide RNA, enabling small insertions, deletions, and base-to-base conversions at a target locus (for example, at MSTN), again without a double-strand break. Epigenetic editing tools, by contrast, do not alter the underlying DNA sequence at all: catalytically dead Cas9 fused to transcriptional activators or repressors (or to DNA methyltransferases/demethylases) is used to up- or down-regulate endogenous gene expression, for instance silencing MSTN transcription without introducing any foreign coding sequence. Because epigenetic editing leaves no mutated or foreign sequence to detect directly, and because base and prime editing leave a smaller genomic footprint than gene addition or double-strand-break-dependent knockout, all three tools are expected to shift the detection burden further toward the indirect, expression- and chromatin-based signatures discussed in Section 5.2, and to complicate the distinction between a naturally occurring genetic variant and an edited one. A summary of the principal gene-doping targets, their mechanisms, and detection approaches is provided in Table 1.

**Table 1:** Principal gene-doping targets: mechanism, performance-relevant effect, delivery, and detection approach.

Target/gene	Mechanism	Performance-relevant effect	Typical vector/delivery	Detection approach
EPO (erythropoietin)	Stimulates erythropoiesis via EPO receptor signalling	Increased red-cell mass and oxygen-carrying capacity (endurance)	AAV, adenovirus, plasmid	Direct: vector/transgene PCR; Indirect: Athlete Biological Passport
MSTN pathway (myostatin/activin inhibition; e.g., follistatin)	Blocks TGF-β superfamily signalling that restrains muscle growth	Muscle hypertrophy/hyperplasia; altered connective tissue	AAV, adenovirus	Direct: vector/transgene PCR (e.g., rAdV-hFST assays); Indirect: expression signatures
IGF-1 and related growth factors (VEGF, HGF)	Promotes myogenesis, angiogenesis, tissue regeneration	Faster muscle repair; trophic effect on muscle mass	AAV, plasmid, electroporation	Direct: transgene detection; Indirect: omics profiling
PPAR-δ/AMPK pathway	Shifts muscle fibre phenotype toward oxidative, fatigue-resistant type	Improved endurance capacity (“exercise in a pill”)	Viral vector (preclinical); pharmacological analogues also studied	Indirect: metabolic/transcriptomic signatures (no validated human assay)
Genome-editing agents (CRISPR-Cas et al.)	Direct editing or epigenetic modulation of endogenous genes (e.g., MSTN)	Potentially any of the above, without exogenous transgene	Ribonucleoprotein, viral or non-viral delivery	Indirect: off-target/edit signatures under development; no validated assay

AAV, adeno-associated virus; VEGF, vascular endothelial growth factor; HGF, hepatocyte growth factor; PCR, polymerase chain reaction.

Sources (1–4, 7, 8, 11, 12).

## 4 Delivery systems and vectors

Most experimental and clinical gene-transfer work relevant to this discussion uses viral vectors [principally adeno-associated virus (AAV) and adenovirus] chosen for their ability to transduce post-mitotic tissues such as skeletal muscle with sustained transgene expression (11, 12). AAV vectors are of particular concern to anti-doping scientists because of their low immunogenicity relative to adenovirus, long-term expression profile, and the existence of approved AAV-based gene therapies (e.g., for spinal muscular atrophy) that demonstrate real-world feasibility of the underlying technology, even though these products are not designed or approved for performance enhancement. Non-viral methods (plasmid DNA, lipid nanoparticles, electroporation-assisted delivery) are less efficient for sustained expression but are simpler to obtain and administer outside clinical settings, which has made them a specific focus of forensic and detection research despite their comparatively modest efficacy.

These vector classes differ enough in their biology, and in the detection strategies they favour, to warrant direct comparison (Table 2).

**Table 2:** Comparison of principal gene-transfer vector types relevant to gene-doping risk and detection.

Vector	Advantages	Disadvantages	Detection implications
AAV (serotypes, e.g., AAV1/2/8/9)	Low immunogenicity; non-integrating (predominantly episomal); long-term transgene expression in post-mitotic tissue (muscle); multiple approved human products demonstrating real-world feasibility.	Limited packaging capacity (∼4.7 kb); pre-existing neutralising antibodies in a substantial minority of the population restrict re-dosing and some serotypes; manufacturing at high purity is demanding.	Durable episomal DNA makes vector-/transgene-specific PCR the most established direct-detection route, but very low copy number and progressive dilution over time still challenge sensitivity.
Adenovirus	Large packaging capacity; very efficient transduction; rapid onset of expression; inexpensive to produce at scale.	Strongly immunogenic (a documented cause of fatal systemic inflammatory response in early clinical gene therapy); transient (non-integrated, episomally lost) expression; widespread pre-existing immunity.	High immunogenicity and transience narrow the detection window but also make covert, repeated self-administration clinically risky; vector-specific PCR assays for recombinant adenoviral constructs (e.g., encoding follistatin) have already been validated in animal models.
Lentivirus	Stable, integrating, long-term expression; can transduce both dividing and non-dividing cells.	Genomic integration carries a theoretical insertional-mutagenesis/oncogenesis risk; more complex manufacturing; less clinical track record in musculoskeletal indications than AAV.	Integrated proviral sequences are, in principle, permanently detectable in transduced cells, but this requires tissue (rather than blood) sampling in most scenarios, limiting practical anti-doping utility outside biopsy-based confirmatory testing.
Plasmid DNA	Simple and cheap to produce; no viral packaging constraints; minimal immunogenicity; does not require specialised virology facilities to manufacture.	Low transduction efficiency; transient expression without repeated dosing or an electroporation-type delivery aid.	Comparatively easy to obtain outside clinical settings, so it is a specific focus of forensic detection research despite its modest efficacy; plasmid backbone sequences (e.g., promoter/vector junctions) provide a distinguishable PCR target from endogenous DNA.
Lipid nanoparticles (LNPs)	Non-viral; low immunogenicity; established manufacturing pipeline following widespread clinical use for mRNA delivery; repeat dosing feasible.	Predominantly deliver transient mRNA rather than sustained DNA-encoded expression; tissue targeting beyond the liver remains technically demanding; formulation stability requires cold-chain handling.	Because LNP-mRNA delivery leaves no integrated or episomal DNA, direct detection is confined to a short window of transcript/protein overexpression, making this modality among the hardest to detect directly and increasing reliance on indirect, omics-based signatures (Section 5.2).

## 5 Detection strategies

Detecting gene doping is substantially more difficult than detecting protein-based doping because the administered material (a nucleic acid sequence or vector) may be present only transiently or at very low copy number in accessible body fluids, and because the resulting gene product is, by design, biologically identical or near-identical to the endogenous protein it mimics (7).

### 5.1 Direct detection

Direct approaches aim to identify the foreign genetic material itself (for example, vector-specific DNA sequences, transgene constructs, or plasmid backbones) typically using polymerase chain reaction (PCR)-based assays such as quantitative real-time PCR (qPCR) or digital droplet PCR applied to blood, plasma, or increasingly to minimally invasive matrices such as dried blood spots, which offer logistical advantages for sample storage and transport in out-of-competition testing (12). WADA-funded research groups have published proof-of-concept assays capable of detecting recombinant adenoviral and AAV vector sequences encoding targets such as follistatin (an endogenous myostatin antagonist) and erythropoietin in animal models, providing a template for assays intended for eventual use in human doping control (11, 12).

### 5.2 Indirect detection

Because direct detection depends on the diminishing window during which vector DNA remains detectable, indirect strategies examine the downstream physiological consequences of gene doping (abnormal patterns of gene expression, characteristic transcriptomic or proteomic “signatures,” or biomarker profiles inconsistent with natural physiology (7). This approach parallels, and can be integrated with, the Athlete Biological Passport model already used to flag physiologically implausible longitudinal blood or steroid profiles, extending the same principle of monitoring an individual's own biological baseline over time rather than searching for a single prohibited substance (5).

### 5.3 Ongoing challenges

Key limitations include the short persistence of episomal or non-integrated vector DNA at the injection site or in circulation, the difficulty of distinguishing a low-dose or localised gene-doping event from background noise, the technical demands of validating assays without a real-world positive reference population, cost and turnaround time for advanced molecular assays relative to routine doping-control testing, and the continual emergence of new vector chemistries and delivery routes that require assay updates. Analytical reviews of the field consistently note that this remains one of the most resource-intensive and rapidly evolving branches of doping-control science (7).

Omics-based strategies are increasingly proposed as a practical complement to targeted PCR and Athlete Biological Passport approaches. Transcriptomic, proteomic, and epigenomic (“multi-omics”) profiling of blood or dried blood spots can in principle detect the downstream signature of a gene-doping event—an abnormal expression pattern across dozens or hundreds of genes, even when the causative construct itself is unknown or no longer detectable, and is analytically agnostic to whether the underlying manipulation was gene addition, base/prime editing, or epigenetic editing (Section 3.5). This generality is also its principal practical limitation: multi-omics assays currently require more sample material, more specialised instrumentation (mass spectrometry, next-generation sequencing), and substantially higher per-sample cost than routine chromatographic or immunoassay-based doping controls, and normal biological and analytical variability across laboratories, populations, and testing conditions makes establishing a validated “abnormal” reference range difficult. These cost and infrastructure demands are a particular constraint for out-of-competition testing, which relies on unannounced sampling at training sites with limited access to laboratory-grade cold-chain logistics and rapid turnaround; scaling omics-based detection to routine out-of-competition use will likely require cheaper, more field-deployable sampling (e.g., further development of dried blood spot workflows) before it can complement, rather than merely supplement in specialist cases, existing detection strategies.

## 6 Beyond genes: microbiome-based performance enhancement and “Faecal doping”

Alongside gene-based approaches, a second and more recently recognised frontier of biological performance enhancement concerns the gut microbiome. Unlike host DNA, the microbiome is a transferable, modifiable ecosystem: whole microbial communities can be transplanted between individuals via faecal microbiota transplantation (FMT), an established clinical procedure for recurrent *Clostridioides difficile* infection and an investigational one for inflammatory bowel disease and metabolic disorders (13). This transferability, combined with growing evidence that elite athletes harbour a distinctive gut microbiota, has raised the prospect of “faecal doping” (termed, perhaps more evocatively, “microbiome doping” in the popular press): transplanting microbiota from high-performing donors, or targeted microbial strains derived from them, into other athletes to confer a performance advantage (14).

This shift in focus reflects a broader conceptual reframing in biology: the athlete, like other multicellular organisms, is increasingly understood not simply as a human genome but as a “holobiont”, a composite biological unit comprising the host together with its associated microbial communities, whose combined genetic and metabolic capacities (sometimes termed the “hologenome”) jointly shape the host phenotype (15, 16). Viewed through this lens, microbiome-based performance enhancement is not a phenomenon separate from gene doping but a second route into the same extended biological system: because part of the holobiont's genetic and metabolic repertoire resides in transplantable microbial cells rather than in the host's own chromosomes, altering that microbial compartment can produce host-level phenotypic effects (such as the lactate-clearing capacity discussed below) that are functionally analogous to those sought through direct manipulation of the host genome.

Three terms recur in this section and are used with distinct meanings throughout. *Microbiota transplantation* (faecal microbiota transplantation, FMT) refers to the clinical/experimental procedure itself: the transfer of a donor's whole microbial community, or a defined subset of it, into a recipient. *Faecal doping* is used here specifically for the hypothetical performance-motivated application of that procedure (i.e., FMT performed with a high-performing donor and a performance rather than medical rationale). *Microbiome doping* is used as the broader umbrella term, encompassing faecal doping as well as narrower strategies such as single-strain probiotic supplementation; it is the term more commonly encountered outside the specialist literature. None of these terms currently correspond to a formally defined WADA prohibited-method category (Section 6.4), and their use in this review should be read as descriptive rather than as asserting an established regulatory classification.

The evidence reviewed in Sections 6.1–6.3 spans several distinct levels that should not be treated as interchangeable. Observational human studies (Section 6.1) establish that athlete and sedentary microbiota differ, but association alone does not establish that the athlete-associated profile causes enhanced performance. Preclinical mouse studies (Sections 6.2–6.3) establish biological plausibility and, in some cases, a defined causal mechanism, but effects demonstrated in mice, even when triggered by human-derived material, do not automatically transfer to human athletic performance. Human intervention studies (Section 6.2) provide the most direct evidence but, to date, have measured fatigue-related and self-reported activity outcomes rather than validated measures of physiological exercise capacity or competitive performance. Distinguishing these levels explicitly is essential to avoid overstating the current evidence base for microbiome doping, which at present should be regarded as biologically plausible but not demonstrated to enhance human athletic performance.

### 6.1 Athlete-associated gut microbiota signatures

Comparative microbiome studies have repeatedly reported that elite athletes and highly active individuals differ from sedentary controls in gut microbial composition and function, including greater microbial diversity, enrichment of taxa such as *Akkermansia muciniphila* and members of the genus *Veillonella*, elevated faecal short-chain fatty acid concentrations, and metabolic pathway profiles skewed toward amino-acid and carbohydrate catabolism (17, 18). Subsequent matched-cohort studies in different athletic populations have found similar, if not always identical, taxonomic differences (for example, enrichment of *Clostridiaceae* and reduction of inflammation-associated taxa such as *Bilophila* in athletes compared with inactive controls), suggesting that exercise-associated microbiota remodelling is a broadly reproducible, though heterogeneous, phenomenon rather than a feature specific to a single sport or cohort (19).

This body of comparative evidence has since been synthesised in a comprehensive narrative review spanning several dozen studies comparing athletes and physically active individuals with sedentary controls, which concluded that exercise, associated dietary factors, and athletic classification collectively promote a more “health-associated” gut microbiota: a higher abundance of health-promoting bacterial species, increased microbial diversity, greater functional metabolic capacity and microbial-associated metabolites (particularly short-chain fatty acids), stimulation of bacterial taxa capable of modulating mucosal immunity, and improved gastrointestinal barrier function (20). This review also emphasises that functional profiling, metagenomic and metabolomic readouts of what the microbiota *does* rather than taxonomic composition alone, is likely to be a more informative and reproducible marker of the athletic phenotype than compositional diversity *per se*, a distinction that mirrors the emphasis in Barton and colleagues’ metagenomic re-analysis of the same rugby cohort discussed above.

### 6.2 *Veillonella atypica* and the lactate–propionate pathway

The most mechanistically developed example of this literature is a 2019 study that profiled the gut microbiota of Boston Marathon runners before and after competition and, separately, a cohort of elite and Olympic athletes, finding a consistent post-exercise enrichment of *Veillonella* (21). The investigators isolated *Veillonella atypica* from athlete stool, administered it to mice, and observed a measurable increase in exhaustive treadmill running time compared with control bacteria. Mechanistic follow-up showed that *Veillonella* uses lactate, the metabolite that accumulates during strenuous exercise, as its principal carbon source, converting it via a defined metabolic pathway into propionate, a short-chain fatty acid; shotgun metagenomic sequencing confirmed that genes in this lactate-to-propionate pathway were more abundant after exercise in the athlete cohort, and direct rectal administration of propionate reproduced the improvement in running performance seen with bacterial supplementation (21). This work has since been extended: a more recent randomised human study, currently available only as a preprint and not yet peer-reviewed, with a mechanistic mouse follow-up, reported that oral *V. atypica* supplementation reduced perceived fatigue and increased voluntary physical activity, with corroborating results in mice. Because these outcomes are fatigue-related and self-reported rather than direct, validated measures of physiological exercise capacity or competitive performance, this study strengthens the case for a causal (rather than merely correlational) effect of this microbial pathway on perceived fatigue specifically, but it does not yet constitute evidence of enhanced exercise performance (22). A smaller, earlier, peer-reviewed pilot study provides some independent human precedent for this line of work, though with a different, more modest result: in a 14-day randomised crossover trial of seven physically active adults, *V. atypica* supplementation was associated with maintained treadmill anaerobic capacity relative to a non-significant declining trend on placebo, but exhaustion time itself and post-exercise lactate did not differ significantly between arms (23). Given its small sample size and the absence of a significant primary endpoint, this study should be read as tolerability and proof-of-concept data rather than as independent confirmation of the fatigue and voluntary-activity effects reported by Aras-Hira et al.

### 6.3 From probiotic supplementation to faecal microbiota transplantation

Two escalating strategies for exploiting this biology can be distinguished. The first, targeted probiotic or single-strain supplementation with organisms such as *V. atypica*, or with commercially available strains such as *Bifidobacterium animalis subsp. lactis* (shown in one quasi-experimental study to reduce a stress-related “choking” phenomenon in elite divers, in parallel with changes in gut Veillonellaceae abundance), is mechanistically modest and closely resembles conventional sports-nutrition supplementation (24). The second, more far-reaching strategy is whole-community FMT from a high-performing donor into a recipient athlete, intended to transfer the donor's entire performance-associated microbial ecosystem rather than a single isolated strain. Commentators in the endocrinology and sports-science literature have explicitly raised this possibility, coining the term “faecal doping” to describe the use of FMT to enhance exercise performance, by direct analogy with blood doping and gene doping, given that regular physical activity is already known to reshape the recipient's own microbiota and that FMT is an established (if still incompletely standardised) technique in clinical gastroenterology (14, 21).

Direct empirical support for the whole-community FMT strategy has since emerged from a study that profiled a cohort spanning a wide range of aerobic exercise capacities and found, somewhat counterintuitively, that gut bacterial *α*-diversity, density, and functional richness were *reduced* rather than increased in athletes with very high exercise capacity. When this atypical, less diverse microbiota was transplanted via FMT from human donors into antibiotic-pretreated mice, recipient animals showed improved insulin sensitivity and greater muscle glycogen stores, with the magnitude of the metabolic benefit tracking the donor's aerobic exercise capacity (25). This finding both complements and diverges from the *Veillonella*-lactate-propionate mechanism described above: whereas Scheiman and colleagues identified a single performance-enhancing taxon acting through a defined metabolic pathway to extend running endurance (21), Martin and colleagues show that transfer of an athlete's entire donor ecosystem, even one of reduced compositional diversity, can confer a metabolic advantage (improved glucose handling and glycogen storage) not attributable to any single isolated strain (25). Read together, the two studies strengthen the plausibility of “faecal doping” from complementary angles: Scheiman et al. supply a mechanistic rationale for single-strain supplementation, while Martin et al. supply direct proof-of-principle that whole-community FMT from high-capacity athletic donors is sufficient to transfer a favourable metabolic phenotype to a recipient organism. It is important to be precise about what this proof-of-principle covers: the demonstrated endpoints were improved insulin sensitivity and increased muscle glycogen storage in recipient mice, not an increase in exercise performance itself, and the transplantation was from human donors into mice rather than between humans. Whether whole-community FMT would reproduce these metabolic effects, let alone translate them into a measurable performance advantage, in a healthy human athlete recipient remains untested. “Faecal doping” based on whole-community FMT should therefore be regarded, at present, as a biologically plausible but unproven performance-enhancement strategy rather than an established one.

### 6.4 Regulatory status and detection challenges

Microbiome-based enhancement occupies a genuine regulatory grey zone. WADA's current Prohibited List does not explicitly name microbiota transfer or FMT as a prohibited method, and it remains unsettled in the anti-doping literature whether such practices would fall under the broad “gene and cell doping” category (given that FMT transfers living cells and their genetic material), under a future dedicated category, or outside the Prohibited List altogether if performed for a legitimate, medically supervised gastrointestinal indication (2, 14). Detection is similarly undeveloped relative to gene doping: because the transplanted organisms are naturally occurring human commensals rather than foreign DNA sequences or synthetic molecules, distinguishing an athlete's exercise-induced enrichment of *Veillonella* or other taxa from a deliberately transplanted, donor-derived microbial community would likely require donor–recipient strain-level metagenomic comparison, longitudinal microbiome monitoring analogous to the Athlete Biological Passport, or identification of an atypical, abrupt shift in microbial community structure inconsistent with the athlete's own training-related trajectory. No validated anti-doping assay for FMT-based enhancement has yet been published, making this an area explicitly flagged for future research. Key evidence on athlete-associated gut microbiota and performance is summarised in Table 3.

**Table 3:** Key evidence on athlete-associated gut microbiota and performance.

Study/organism	Population/model	Key finding	Proposed mechanism
Clarke et al. (17)	Elite athletes vs. sedentary controls (human)	Greater gut microbial diversity; enrichment of Akkermansia and Veillonella in athletes	Exercise- and diet-associated remodelling of gut ecosystem
Barton et al. (18)	Professional athletes vs. sedentary subjects (human)	Distinct compositional and functional (metabolic) microbiome profile in athletes	Enhanced amino-acid/carbohydrate metabolic pathway capacity
Scheiman et al.(21) (Nat Med)	Boston Marathon runners; elite/Olympic athletes; mouse gavage	Post-exercise Veillonella enrichment; V. atypica improved mouse treadmill run time ∼13%	Lactate-to-propionate conversion via Veillonella metabolic pathway
2025 RCT + mouse validation (preprint)	Human RCT with mechanistic mouse follow-up	Oral V. atypica supplementation reduced fatigue interference, increased voluntary activity	Propionate-mediated reduction in exercise-associated fatigue signalling
Dong et al. (24)	Elite diving athletes (human, quasi-experimental)	B. animalis subsp. lactis BB-12 yogurt reduced stress-related “choking” phenomenon	Modulation of gut Veillonellaceae abundance
Martin et al. (25) (Cell Rep)	Human donors across exercise-capacity gradient; FMT into mice	Reduced gut microbial diversity in very-high-capacity athletes; donor microbiota engraftment improved recipient-mouse insulin sensitivity and muscle glycogen stores	Whole-community FMT effect on host glucose/glycogen metabolism, independent of any single strain
Matched-cohort studies [e.g., Xu et al. (19)]	Elite athletes vs. active/inactive young adults (human)	Enrichment of Clostridiaceae; reduction of inflammation-linked taxa (e.g., Bilophila) in athletes	Activity-associated shift away from pro-inflammatory microbial profile

RCT, randomised controlled trial. Findings are drawn from human observational/small-intervention studies and mouse models; causal translation to human athletic performance beyond the cited studies remains an active research question. The Aras-Hira et al. (22) entry is a preprint, not yet peer-reviewed, and its outcomes (fatigue, voluntary activity) are distinct from validated measures of exercise capacity or competitive performance; the Martin et al. (25) entry reports metabolic outcomes in recipient mice and has not been demonstrated to transfer to human recipients or to enhance exercise performance. Sources (17–19, 21–25).

## 7 A third frontier: anti-aging gene therapy and its relevance to sports rehabilitation

A third, closely related frontier concerns gene-based interventions designed not to treat a specific injury or disease but to slow, halt, or partially reverse biological aging (26). This field is of direct relevance to sports rehabilitation because its principal targets (skeletal-muscle mass and regenerative capacity, connective-tissue quality, and stem-cell function) are precisely the tissues and processes that determine how well an aging or master athlete recovers from injury, and because several of its candidate genes (notably follistatin) are the same ones already discussed above as gene-doping targets, further blurring the line between anti-aging medicine, regenerative rehabilitation, and prohibited enhancement.

### 7.1 Rationale: aging, sarcopenia, and athletic longevity

Skeletal muscle mass, strength, and regenerative capacity decline progressively with age, a process termed “sarcopenia”, and this decline is a major determinant of both athletic longevity in older competitors and functional recovery after injury or surgery later in life (26). Because rehabilitation outcomes in older patients are strongly shaped by the underlying biological, rather than merely chronological, age of muscle, tendon, and stem-cell compartments, interventions capable of restoring a more youthful regenerative phenotype are of obvious interest to sports medicine and rehabilitation, independent of any application in uninjured, high-performance athletes.

### 7.2 Key molecular targets

Three lines of research dominate current anti-aging gene therapy. First, telomerase (TERT) gene delivery aims to lengthen or maintain telomeres (the protective DNA caps that shorten with each cell division and are used as a biomarker of cellular aging) with the stated goal of extending the replicative capacity of stem and somatic cells. Second, Klotho, an endogenous “anti-aging” hormone that declines with age and inhibits several pro-aging signalling pathways (TGF-β, IGF-1, Wnt, and NF-κB) (26), has been delivered via AAV vectors in aged wild-type mice. Intraventricular AAV-mediated expression of secreted and transmembrane Klotho isoforms in the senescence-accelerated SAMP8 model improved cognitive performance, physical state, and bone microstructure relative to age-matched controls (27). More recently, systemic AAV9-mediated delivery of secreted Klotho in aged wild-type mice produced a 20% extension in remaining lifespan alongside improvements in physical fitness, reduced muscle fibrosis, increased muscular regenerative capacity, and improved bone microstructure, in a single-dose study published in 2025—findings of particular interest to sports rehabilitation given the direct musculoskeletal benefit observed (28). Third, partial cellular reprogramming using a subset of the Yamanaka transcription factors (OCT4, SOX2, and KLF4, with or without c-MYC; OSK/OSKM) can reset markers of cellular age without erasing cell identity; systemically delivered AAV-OSK in aged mice has been reported to more than double median remaining lifespan and improve frailty scores (29), while tissue-restricted expression of the same factors directly in muscle fibres has been shown to reactivate quiescent muscle stem cells and accelerate muscle regeneration after injury (30), making this line of research a particularly direct bridge between anti-aging biology and musculoskeletal rehabilitation science.

### 7.3 Self-experimentation and unregulated clinical use

Unlike most gene-doping targets, which remain largely theoretical in humans, anti-aging gene therapy has already moved into unregulated human self-experimentation. The most widely reported case is that of a biotechnology-company founder who, outside any approved clinical trial, received her own company's experimental gene therapies encoding telomerase and follistatin, reporting subsequent telomere lengthening in blood cells (31, 32); the same company has since filed patents covering AAV-delivered TERT and Klotho gene therapy administered via injectable or intranasal routes for longevity, cognitive, and musculoskeletal indications (33). This patent rests on scientifically contested ground: a peer-reviewed study describing the same intranasal/injectable TERT- and follistatin-based platform, co-authored by the patent holder, was retracted by the journal in 2025 after an institutional review identified data discrepancies (34), a fact that underscores the need for caution in treating this patent as evidence of validated therapeutic efficacy. A small but active “longevity clinic” and biohacker ecosystem now offers gene-therapy-based interventions outside the framework of national medicines regulators. This pattern (self-administration or direct-to-consumer provision of unapproved gene constructs targeting muscle and connective-tissue aging) mirrors, and in practice may directly overlap with, the unregulated acquisition pathways of concern in classical gene doping, and is highly relevant to master and veteran athletes, who represent a plausible early-adopter population for such interventions.

Much of the material in this subsection derives from science journalism, company patents, and other non-peer-reviewed sources rather than from peer-reviewed primary research, reflecting the fact that self-experimentation of this kind is conducted outside the peer-reviewed clinical-trial literature. These sources are used here because they are, at present, the only available documentation of this practice, but they should be interpreted with corresponding caution: they are not subject to independent editorial or scientific review, may reflect promotional interests, and cannot substitute for peer-reviewed efficacy or safety data. Readers should treat the human “evidence” summarised here as documentation that self-administration has occurred, not as validated evidence of therapeutic or performance benefit.

### 7.4 Regulatory status and anti-doping relevance

Anti-aging gene constructs targeting muscle mass, regenerative capacity, or metabolic function (including follistatin- and myostatin-pathway agents, and potentially TERT or OSK-based products should they demonstrate musculoskeletal performance effects in humans) would fall squarely within the broad language of WADA's Section M3 if used by a competing athlete with the intent or effect of enhancing performance, regardless of whether the product is marketed under a longevity or anti-aging label rather than a sport-performance one (2). This creates an additional practical detection and governance challenge: an athlete could, in principle, obtain a nominally “anti-aging” gene therapy through a longevity clinic or self-experimentation network without ever describing the intervention in doping-related terms, complicating both self-disclosure at doping control and public-health tracking of the underlying practice. As with gene and microbiome doping, to the author's knowledge, no confirmed case of anti-aging gene therapy use for sport-performance purposes has been publicly documented as of the time of writing, but the existence of real, marketed, self-administered products in this space makes it a nearer-term practical concern than some other theoretical gene-doping scenarios. Anti-aging gene-therapy targets, their evidence base, and their relevance to rehabilitation are summarised in Table 4.

**Table 4:** Anti-aging gene-therapy targets: mechanism, evidence base, key risk, and relevance to rehabilitation.

Target	Mechanism	Evidence base (reported human use vs. efficacy)	Key risk	Relevance to rehabilitation
Telomerase (TERT)	Lengthens/maintains telomeres to extend replicative capacity of cells	Efficacy evidence: animal models only. Reported human use: one self-administered case (not an efficacy trial)	Theoretical oncogenic potential from constitutive activation	Potential stem-cell/tissue-repair capacity in aging patients (unproven in humans)
Klotho (KL)	Inhibits TGF-β, IGF-1, Wnt and NF-κB pro-aging pathways	AAV delivery in aged mice: extended lifespan, improved fitness, muscle regeneration, bone microstructure	Long-term human safety largely uncharacterised	Direct musculoskeletal regenerative benefit relevant to sarcopenia and injury recovery
Partial reprogramming (OSK/OSKM; Yamanaka factors)	Resets epigenetic/cellular age markers without erasing cell identity	AAV-OSK extended remaining lifespan in aged mice; myofiber-restricted OSKM accelerated muscle regeneration	Teratoma formation/loss of cell identity if expression is not tightly time-limited	Direct bridge to muscle-stem-cell (satellite cell) regenerative therapy
Follistatin (cross-reference: MSTN pathway)	Antagonises myostatin/activin signalling	Reported human use (one self-administered case; not an efficacy trial); extensive preclinical muscle-hypertrophy data	Overlaps directly with classical gene-doping risk profile (Table 1)	Same biology proposed for sarcopenia and age-related muscle wasting

AAV, adeno-associated virus. OSK/OSKM = Oct4/Sox2/Klf4(/c-Myc) partial-reprogramming factor set. Human musculoskeletal efficacy data for TERT, Klotho, and OSK-based approaches remain limited to a single self-administered case and animal models respectively. Sources (4, 8, 26, 28–30, 32, 33).

## 8 The dual-use dilemma: legitimate regenerative, microbiome-directed, and anti-aging therapies in sports rehabilitation

A distinctive feature of gene doping, compared with most other doping categories, is how closely its candidate agents overlap with legitimate regenerative medicine research aimed at exactly the injuries that sports rehabilitation clinicians treat every day. Gene-transfer strategies delivering growth factors such as bone morphogenetic proteins, IGF-1, or vascular endothelial growth factor to bone, cartilage, tendon, and ligament have been investigated since the late 1990s as a way to overcome the intrinsically limited healing capacity of these tissues (3), and more recent work has combined gene-loaded tendon grafts or scaffolds with reconstructive surgery to improve tendon and ligament repair in animal models, alongside complementary biologic approaches (e.g., platelet-rich plasma, mesenchymal stem cells) under investigation for the same indications (35). Myostatin blockade, discussed above as a doping concern, is simultaneously being studied as a strategy to counter the quadriceps atrophy and weakness that persist in many patients even after adequate rehabilitation following ACL injury (36). Anti-aging gene therapy sharpens this dilemma further still, because interventions such as Klotho delivery or muscle-targeted partial reprogramming are explicitly being developed to restore youthful regenerative capacity in aging tissue (27, 28, 30), precisely the outcome a sports-medicine clinician would want for an older patient recovering from injury, and precisely the outcome that would be prohibited if pursued by a competing master or veteran athlete for the purpose of enhancing performance.

This convergence creates a genuine governance challenge. A gene-based intervention intended to restore normal tissue function and expedite an athlete's safe return to sport after injury may be biologically indistinguishable, at the molecular level, from an intervention intended to enhance performance beyond the individual's natural baseline. The distinction often rests on intent, dose, and whether the treated tissue or system was pathological or already healthy, rather than on the biology of the vector itself. The same logic extends to the microbiome and to anti-aging biology: FMT is a legitimate, guideline-supported treatment for recurrent *C. difficile* infection and is under active clinical investigation for inflammatory bowel disease and metabolic conditions relevant to athletes (13), yet the identical procedure performed with a high-performing donor and a performance rationale would be widely regarded as a doping practice in the informal, commonly understood sense, even though, as discussed in Section 6.4, it is not yet formally classified as a prohibited method under the current WADA Prohibited List; similarly, a follistatin- or Klotho-based therapy prescribed to counter genuine age- or injury-related muscle wasting sits on the same biological continuum as its use to enhance an otherwise healthy older athlete's competitive performance. Rehabilitation professionals and sports physicians should therefore be alert to: (i) the regulatory and off-label status of any gene-based, cell-based, microbiota-based, or anti-aging product or procedure offered to an athlete; (ii) the importance of therapeutic indication and documentation when growth-factor, regenerative, microbiome-directed, or anti-aging techniques are used in an athlete population, particularly older or master athletes for whom the treatment-vs.-enhancement distinction is least clear; and (iii) the reputational, legal, and anti-doping-rule-violation risk that can arise even from well-intentioned use of emerging biologics without appropriate authorisation, clinical trial oversight, or, where applicable, a Therapeutic Use Exemption.

## 9 Safety and health risks

Beyond anti-doping considerations, unregulated or self-administered gene transfer carries substantial direct health risks. Historical adverse events in the gene-therapy field, including immune-mediated toxicity and, in early trials using earlier-generation adenoviral vectors, a fatal systemic inflammatory response (37), illustrate that vector-related toxicity, insertional mutagenesis, uncontrolled or supraphysiological transgene expression, and unpredictable immune responses are genuine risks even under controlled clinical conditions. An athlete obtaining an unapproved gene-doping product outside a regulated clinical trial would typically lack quality control over vector purity, dosing, and manufacturing, as well as clinical monitoring for the adverse effects described above, compounding these risks considerably (6). Sustained overexpression of erythropoietin, for example, could plausibly increase haematocrit to dangerous levels with associated thrombotic risk, while uncontrolled myostatin-pathway suppression raises theoretical concerns about connective-tissue and cardiac effects that have not been fully characterised in humans.

FMT carries its own, well-documented safety profile that would apply equally to any performance-motivated use. Regulatory agencies have issued formal safety communications after cases in which FMT transmitted multidrug-resistant organisms, including at least one instance associated with a recipient's death, because donor screening protocols failed to detect the resistant organism (38, 39); other recognised risks include transmission of undetected enteric pathogens, unpredictable and potentially long-lasting shifts in recipient metabolic or immune phenotype, and, in the specific context of an ostensibly healthy athlete, the introduction of gastrointestinal symptoms or dysbiosis with no proven benefit outside a controlled research setting. Because FMT performed for enhancement rather than an approved medical indication would fall outside routine clinical governance and donor-screening infrastructure, any such use would plausibly carry a higher risk profile than the same procedure performed in an accredited clinical programme.

Anti-aging gene therapies carry a further, distinct risk profile. Reprogramming-based approaches (OSK/OSKM) are derived from the same transcription-factor cocktail used to generate induced pluripotent stem cells, and continuous or excessive expression of these factors is well known to risk teratoma formation and loss of cell identity *in vivo*, which is why published protocols rely on carefully time-limited or cyclic induction rather than constitutive expression (29, 30), a safety-critical parameter that would be extremely difficult to control outside a specialised research or clinical setting. Long-term human safety data for telomerase-activating or Klotho-overexpressing gene therapies are essentially absent outside the single self-administered case discussed above (32), and constitutive telomerase activation carries a long-recognised theoretical association with oncogenesis given telomerase's role in enabling unlimited replication of malignant cells. As with gene doping generally, self-administration or acquisition through unregulated longevity clinics removes the quality-control, dosing precision, and adverse-event monitoring that would normally accompany such interventions in an approved trial.

## 10 Ethical, legal, and governance considerations

The ethical debate around gene doping mirrors broader discussions about genetic enhancement but is sharpened by the specific values sport claims to protect: fair competition, a level playing field, and athlete health and welfare (40). WADA's Gene and Cell Doping Panel has repeatedly emphasised that anti-doping policy in this area needs to remain connected to wider societal values around genetic technology, rather than being framed purely as a technical detection problem (5). Questions frequently raised in this literature include where genetic “treatment” ends and genetic “enhancement” begins, how the anti-doping system should treat athletes with naturally occurring performance-relevant genetic variants (as opposed to those who have acquired such variants through gene transfer), and how much weight should be given to bodily autonomy vs. the collective interest in preserving comparability of athletic achievement (40).

Microbiome-based enhancement adds a further layer to this debate, because the gut microbiome sits conceptually between “nature” and “training”: it is neither part of an athlete's inherited genome nor simply the product of effort in the way that adaptation to a training programme is usually understood, yet it is also not an external substance in the way a drug is. Whether transplanting another person's performance-associated microbiota should be treated as more akin to blood doping (transferring another individual's biological material for an unearned advantage) or to permitted nutritional strategies (such as probiotic yoghurt or fibre supplementation aimed at shaping one's own microbiota) is an open normative question that anti-doping policy has not yet resolved.

Anti-aging gene therapy raises a related but distinct question: whether restoring a younger biological phenotype in an older athlete should be understood as legitimate treatment of an age-related deficit (analogous to treating any other diagnosed condition) or as enhancement beyond that individual's own natural trajectory, given that aging itself, unlike an acute injury, affects everyone and is not conventionally classified as a disease. This question has particular salience for masters and veteran sport, where age-graded or age-category competition is explicitly built around the assumption of a roughly comparable, natural rate of age-related decline across competitors within a category, an assumption that biologically effective anti-aging gene therapy would directly undermine, arguably more fundamentally than most conventional doping methods, which typically act on otherwise age-independent physiology. For rehabilitation clinicians, these debates translate into concrete practical duties: informed consent processes that clearly distinguish approved from experimental interventions, careful documentation of therapeutic rationale for any gene-, cell-, microbiota-, or anti-aging-based product, and awareness of applicable Therapeutic Use Exemption procedures when a legitimate medical need intersects with a substance or method on, or adjacent to, the Prohibited List.

The discussion above is framed largely in the principle-based terms (fairness, autonomy, harm) that dominate mainstream anti-doping ethics, but complementary frameworks can sharpen the specific dilemmas facing rehabilitation clinicians. A virtue-ethical lens directs attention to the character and practical wisdom of the clinician navigating ambiguous cases, asking not only “is this permitted?” but “what would a conscientious rehabilitation professional do here, given uncertainty?” A care-ethics perspective foregrounds the clinician's relational responsibility to the individual athlete-patient in front of them, which can sit in tension with the population-level, rule-based logic of anti-doping regulation, particularly when an older athlete's genuine medical need for regenerative or anti-aging treatment is constrained by a rule designed to prevent competitive advantage-seeking. Empirical bioethics approaches, which combine normative analysis with qualitative or survey data on how athletes, clinicians, and regulators actually reason about these cases, could help test whether the treatment-vs.-enhancement distinctions drawn in this review track the intuitions of those who must apply them in practice; to our knowledge, no such empirical work yet exists specifically for the microbiome- or anti-aging-doping frontiers, which we identify as a priority for future interdisciplinary research (Section 12).

## 11 Implications for sports rehabilitation practice

Several practical recommendations follow from the material reviewed above for clinicians, physiotherapists, and researchers working in sports rehabilitation:

- Maintain current knowledge of the WADA Prohibited List, particularly Section M3 (Gene and Cell Doping) (2), and of any regenerative, biologic, microbiota-based, or anti-aging therapy's regulatory status before recommending or administering it to an athlete.
- Document the clinical indication, informed consent, and provenance of any growth-factor, cell-based, gene-based, microbiome-directed, or anti-aging product (including probiotics or “longevity” supplements marketed with performance claims) used in an athlete's rehabilitation, to protect both patient and clinician and to support any necessary Therapeutic Use Exemption application.
- Be alert to patients seeking unproven “accelerated recovery,” “performance microbiome,” or “longevity” interventions, including FMT and self-administered or clinic-based gene therapies, obtained outside regulated healthcare or clinical-trial settings and be prepared to discuss the associated health and anti-doping risks candidly, particularly with older and master athletes who may be specifically targeted by longevity-clinic marketing.
- Engage with, or refer athletes to, accredited anti-doping education resources, since athlete-facing education has been identified as a key preventive strategy alongside laboratory detection.
- Recognise that rehabilitation professionals are often among the first point of contact for athletes considering or using emerging biological interventions, particularly in the context of injury recovery or age-related performance decline. Structured education programmes should address the risks of unregulated gene therapy, microbiome manipulation, and anti-aging interventions, and should provide clear guidance on Therapeutic Use Exemption procedures and the regulatory status of emerging biologics; this is particularly important for master and veteran athletes, who may be specifically targeted by longevity-clinic marketing and may not perceive a “wellness” or “longevity” intervention as falling within the scope of anti-doping rules at all.
- Support and participate in interdisciplinary research bridging regenerative sports medicine, microbiome science, aging biology, and anti-doping science, given the shared biological ground across these fields.

These recommendations can be organised into a simple decision framework for a rehabilitation clinician confronted with a specific gene-, cell-, microbiota-, or anti-aging-based intervention for an athlete-patient: (i) *classify* the intervention against the current WADA Prohibited List (explicitly covered under Section M3; plausibly covered by analogy but not explicitly named, as with FMT; or outside its current scope), consulting Sections 2, 6.4, and 7.4 of this review as a starting point; (ii) *assess* whether a genuine, documentable clinical indication exists, distinct from a performance-enhancement rationale; (iii) *determine* whether a Therapeutic Use Exemption is required or advisable given the athlete's competitive status; and (iv) *document* the clinical rationale, informed consent, and provenance of the intervention regardless of the outcome of steps (i)–(iii). I offer this sequence as a starting heuristic rather than a validated clinical tool; formal development and validation of a decision-support instrument along these lines is identified as a priority for future interdisciplinary work in Section 12.

## 12 Future directions

Anti-doping science in this area is likely to continue evolving along several parallel tracks: refinement of direct, vector-specific molecular assays (including minimally invasive sampling such as dried blood spots and increasingly sensitive digital PCR and sequencing methods) for gene doping; expansion of indirect, omics-based and biological-passport-style monitoring capable of flagging physiologically implausible profiles without requiring prior knowledge of the exact construct used; and, for the microbiome, development of strain-level metagenomic and longitudinal monitoring tools capable of distinguishing an athlete's own exercise-induced microbiota shifts from a transplanted, donor-derived community. Anti-aging interventions add a further requirement: biological-age biomarkers such as epigenetic (methylation) clocks, already used in the aging-biology literature to validate the effects of interventions like partial reprogramming, could plausibly be adapted as an indirect anti-doping signal of otherwise undisclosed anti-aging gene therapy, though this application remains speculative and unvalidated for doping-control purposes. WADA and affiliated researchers will also need to clarify, in policy terms, whether and how FMT, TERT/Klotho gene therapy, and partial-reprogramming approaches fall within the existing Prohibited List framework, and whether masters/veteran competition needs bespoke consideration given how directly anti-aging biology could disrupt the assumptions underlying age-graded sport. At the same time, legitimate gene-, cell-, microbiome-, and aging-biology-based regenerative therapies for tendon, ligament, cartilage, muscle, and gastrointestinal health are progressing through preclinical and early clinical research, meaning the sports rehabilitation field will need clear, updated clinical governance frameworks to allow beneficial innovation to reach injured and aging athletes safely while remaining clearly distinguishable, procedurally and documentarily, from prohibited performance enhancement (5, 7).

Translating these research priorities into governance action, I suggest four concrete steps for WADA and affiliated bodies to consider: (i) formally clarify whether performance-motivated FMT falls within the existing “gene and cell doping” category (on the grounds that it transfers living cells and their genetic material) or requires a dedicated category of its own, since the current silence leaves both athletes and clinicians without a clear compliance target; (ii) invest in the development of strain-level metagenomic monitoring, analogous to the Athlete Biological Passport, as the most plausible route to a validated microbiome-doping detection method; (iii) evaluate biological-age biomarkers such as epigenetic (methylation) clocks as an indirect signal of undisclosed anti-aging gene therapy, while explicitly acknowledging that this application remains unvalidated for doping-control purposes and requires dedicated research before any policy use; and (iv) consider whether masters/veteran competition requires bespoke regulatory provisions, given that age-graded sport is structurally more vulnerable to disruption by effective anti-aging biology than most conventional doping methods.

**Limitations.** As a narrative rather than systematic review, this work did not apply a pre-registered search protocol, dual independent screening, or formal risk-of-bias assessment (see the literature-search-strategy paragraph in Section 1), and source selection therefore reflects the author's judgement of relevance rather than an exhaustive or reproducible sampling of the literature; a degree of selection bias cannot be excluded. Much of the mechanistic evidence reviewed, particularly for anti-aging gene therapy and several microbiome-doping pathways, derives from animal models whose translation to human athletic physiology remains unproven (Sections 6 and 7), and a minority of sources are non-peer-reviewed (preprints, patents, news reports), a limitation flagged explicitly at each relevant point in the text. These constraints are inherent to reviewing a fast-moving, still-largely-theoretical field and should be borne in mind when weighing the strength of the conclusions offered below.

## 13 Conclusions

Gene doping remains, as far as public evidence indicates, a largely theoretical threat in competitive sport, though this characterisation should be read against the rapid pace of underlying technological development (base, prime, and epigenetic editing; Section 3.5), which could narrow the gap between theoretical and practical feasibility faster than anti-doping detection capability currently advances; microbiome-based “faecal doping” is, at present, an even earlier-stage concern grounded mainly in mechanistic animal and small human studies rather than confirmed cases of misuse; and anti-aging gene therapy, while still experimental, has already produced at least one well-documented instance of unregulated human self-administration, making it in some respects the most immediately concrete of the three frontiers. All three fields are advancing quickly, and all three overlap substantially with legitimate regenerative, microbiota-directed, or aging-biology approaches relevant to injury recovery and athlete health, particularly for older and master athletes. For the sports rehabilitation community, this means that gene doping, microbiome doping, and anti-aging gene therapy cannot be treated solely as anti-doping laboratory problems: each is also a clinical governance issue, requiring rehabilitation professionals to understand the underlying science, the (in the case of the microbiome and aging biology, still-evolving) regulatory framework, and their own responsibilities when biologic, gene-based, microbiota-based, or longevity-oriented interventions are considered for injured or aging athletes. Continued collaboration between molecular biologists, microbiome and aging researchers, anti-doping scientists, and clinicians will be essential to ensure that genuine therapeutic advances benefit injured athletes without compromising the principles of fair and safe competition. Concretely, this means WADA clarifying the regulatory status of microbiota transfer and anti-aging gene constructs, researchers prioritising strain-level metagenomic and biological-age-biomarker detection methods, and rehabilitation professionals adopting the documentation and Therapeutic Use Exemption practices outlined in Section 11, steps that, taken together, would let clinical governance in this area keep pace with the science rather than perpetually follow it.
